# Successful laparoscopic approach for idiopathic adult colo-colonic intussusception: a case report

**DOI:** 10.1186/s40792-020-01077-4

**Published:** 2020-11-25

**Authors:** Kyota Tatsuta, Mayu Sakata, Kosuke Sugiyama, Toshiya Akai, Katsunori Suzuki, Yuhi Suzuki, Takafumi Kawamura, Yoshifumi Morita, Hirotoshi Kikuchi, Yoshihiro Hiramatsu, Kiyotaka Kurachi, Hiroya Takeuchi

**Affiliations:** Department of Surgery, Hamamatsu University School of Medicine, 1-20-1, Handayama, Higashi-ku, Hamamatsu, Shizuoka 431-3192 Japan

**Keywords:** Idiopathic intussusception, Laparoscopy, Emergency surgery

## Abstract

**Background:**

Adult intussusception is recognized as an abdominal emergency. More than 90% of adult patients with intussusception have distinct causes that are related to benign or malignant tumors. In contrast, idiopathic intussusceptions, which are observed in children, are rare conditions in adult. Especially, colo-colonic idiopathic intussusceptions are rare among them. Surgery is traditionally considered the primary treatment option. Recently, laparoscopic surgery has been reported to be safe and feasible. However, laparoscopic surgical reduction, which is a common procedure in pediatric surgery, is not common in adult intussusception.

**Case presentation:**

We report a 34-year-old man who presented with sudden abdominal pain. Computed tomography revealed the target sign in the transverse colon. There was no evidence of bowel obstruction, bowel wall edema, or tumor. We diagnosed idiopathic intussusception and selected laparoscopic procedure because of the strong abdominal pain experienced by the patient. There were no signs of perforation, bowel wall ischemia, or tumor in abdominal cavity. We confirmed idiopathic colo-colonic anterograde intussusception. Laparoscopic surgical reduction was achieved by a combination of delicate direct pressure on the anal side of the transverse colon and gentle pulling on the oral side. The patient’s postoperative course was uneventful.

**Conclusions:**

We achieved successful surgical reduction laparoscopically because of an accurate preoperative diagnosis based on characteristic computed tomography features and appropriate surgical technique. Laparoscopic procedure and surgical reduction is considered to be an effective treatment strategy for adult idiopathic intussusceptions with severe symptoms.

## Background

Adult intussusception represents 5% of all cases of intussusception and only 1–5% of bowel obstruction cases in adults are due to intussusception [1]. More than 90% of adult patients with intussusception have distinct causes that are related to the small or large intestine. Etiologies of adult intussusception include tumor- or surgery-related, idiopathic, and “other” causes. Benign or malignant tumors are the most frequent cause of intussusception in adults [2, 3]. This is in contrast to 90% of childhood intussusception cases that are idiopathic [4].

Adult idiopathic intussusceptions are rare conditions; therefore, idiopathic cases are considered as the exception [5]. Ileo-colic intussusceptions represent the majority of adult idiopathic cases, whereas colo-colonic idiopathic intussusceptions are rare because many colo-colonic cases are related to primary adenocarcinoma of the colon [2, 3].

Surgery is traditionally considered the primary treatment option and open procedures are often chosen because of bowel obstruction or ischemia [3]. Recently, laparoscopic surgery has been shown to be safe and feasible and has the benefits associated with minimally invasive surgery [6]. Nonetheless, laparoscopic surgical reduction, which is common procedure in pediatric surgery, is not common in adult intussusception because of tumors [2].

We reported the experience with laparoscopic surgical reduction of idiopathic adult colo-colonic intussusception.

## Case presentation

**Fig. 1:**
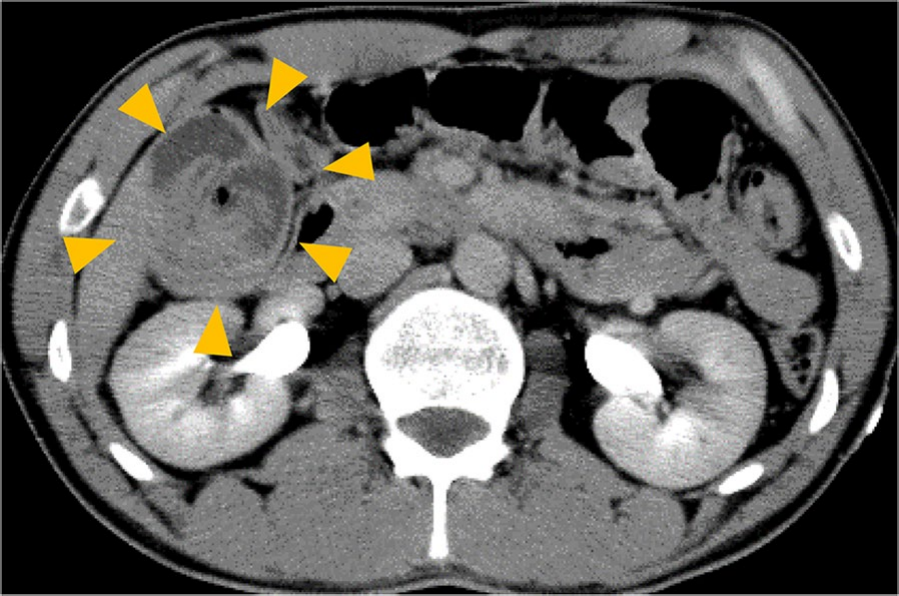
Preoperative contrast-enhanced computed tomography. The target sign was observed in the transverse colon. Distinct anatomic features are clearly seen, such as the entering wall, mesenteric fat and vessels, returning wall, and intraluminal space (yellow arrow)

A 34-year-old man without significant medical history was admitted to our hospital due to upper abdominal pain. Physical examination showed significant abdominal pain. Laboratory data were within normal range, except for a slightly increased C-reactive protein level (0.40 mg/dL). Contrast-enhanced computed tomography (CT) showed the target sign in the transverse colon. There was no evidence of bowel obstruction, bowel wall edema, or tumor (Fig. 1) and idiopathic colo-colonic intussusception was suspected. Because of excruciating pain, he had difficulty maintaining the position for colonoscopy. Therefore, we decided against performing colonoscopic reduction. Laparoscopic surgical reduction was planned to avoid resection of an excessive length of bowel.

**Fig. 2:**
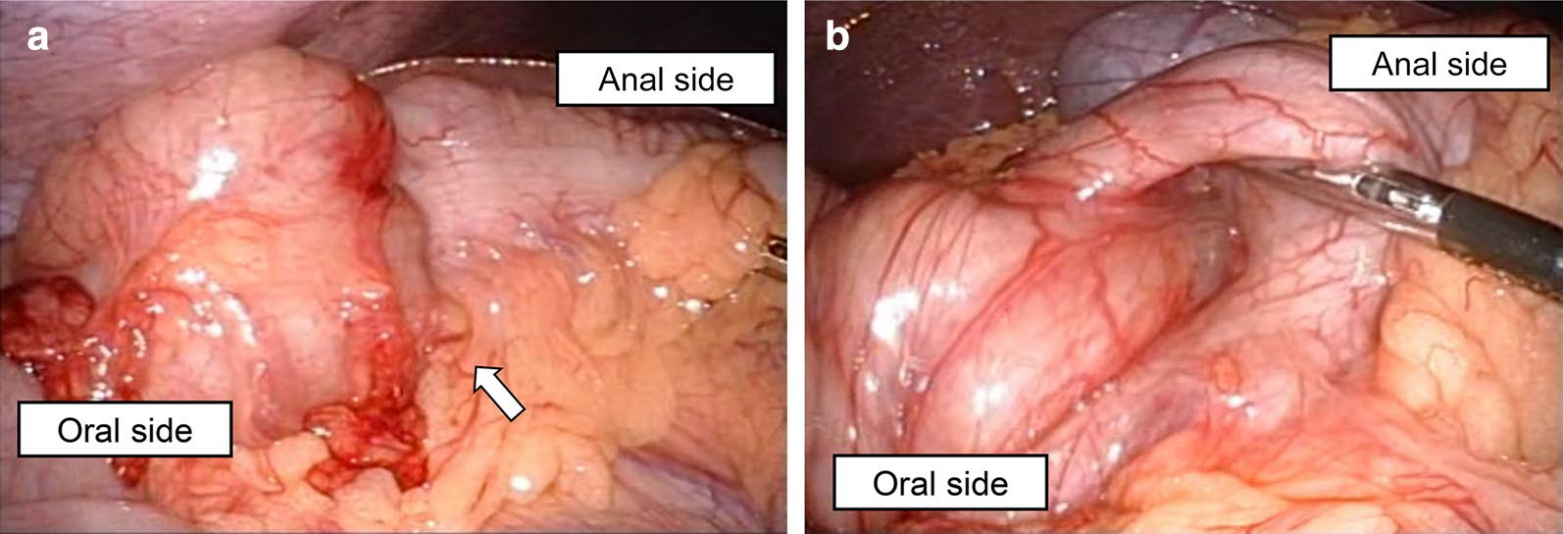
Intraoperative findings. **a** Intussusception was found in the transverse colon (white arrow). **b** Gentle pulling of the bowel on the oral side and horizontally pushing the bowel on the anal side achieved successful reduction

**Fig. 3:**
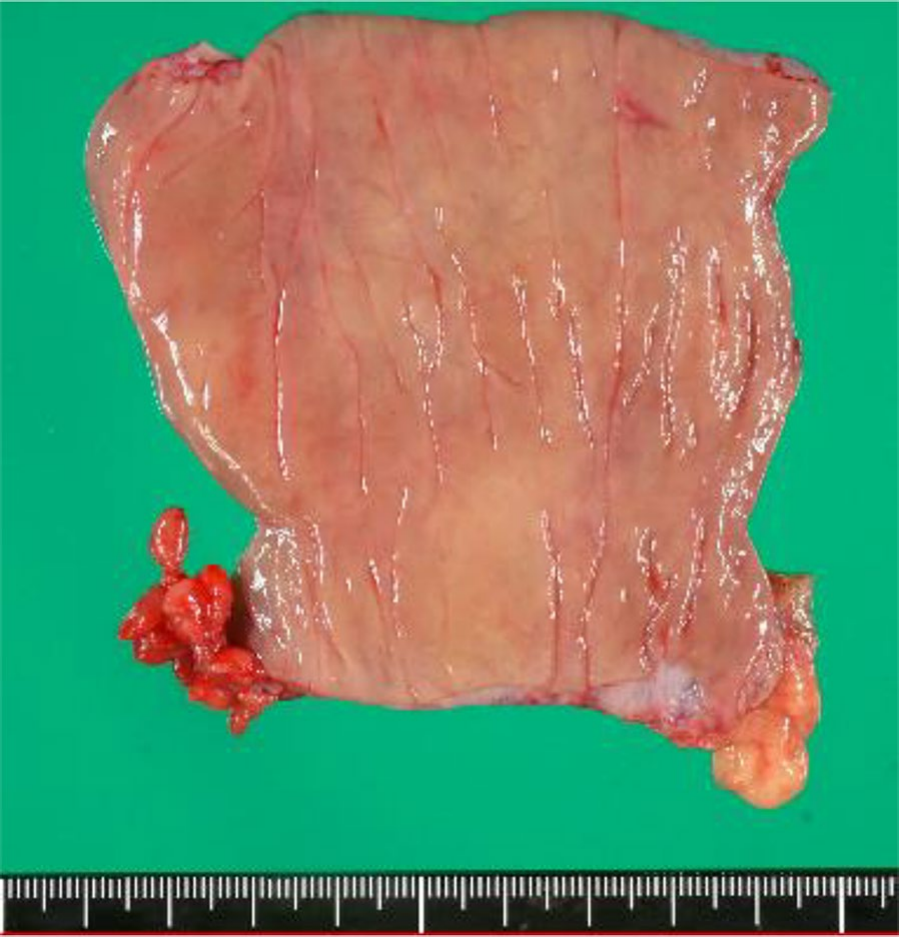
Postoperative specimen. No obvious change was observed on the mucosal surface of the resected bowel

During the laparoscopy procedure, a 12-mm port was inserted at the periumbilical region using the open technique and an intra-abdominal pressure of 10 mm Hg was established with carbon dioxide insufflation. Under laparoscopic observation, additional 5-mm ports were inserted at the lower abdominal quadrants bilaterally and the midline. Thorough inspection of the peritoneal cavity showed no signs of perforation, bowel wall ischemia, or tumor and confirmed idiopathic colo-colonic anterograde intussusception. Surgical reduction was achieved by a combination of delicate direct pressure on the anal side of the transverse colon and gentle pulling on the oral side (Fig. 2). Partial resection of the intussuscepted bowel was performed due to bowel wall edema and Gambee's layer to layer anastomosis was performed. No obvious tumor was observed in the resected bowel on macroscopic examination. Histopathological examination showed significant edema and vasodilation of the submucosa. There was no evidence of special intussusception, such as a parasite. Idiopathic colo-colonic intussusception was confirmed (Fig. 3). The operation time was 131 min, and total blood loss was 20 ml. The patient was discharged on the 7th postoperative day once bowel function returned. Endoscopic observation was performed after discharge, and it was confirmed that there were no other lesions that could lead to colo-colonic intussusception.

## Discussion

Intussusceptions can be classified into three types based on location: (i) enteroenteric, when confined to the small bowel; (ii) colo-colonic, when involving the large bowel; and (iii) enterocolonic, which can be ileocecal or ileocecocolonic. Colo-colonic intussusceptions are the least common type [2, 3, 7]. We achieved successful surgical reduction laparoscopically because of an accurate preoperative diagnosis based on characteristic CT features and surgical technique.

Several imaging modalities can assist in the diagnosis of intussusception, such as abdominal plain radiography, ultrasonography, and CT [8]. Of these, CT is the most accurate, with a reported diagnostic accuracy of 78–100% [8–10]. The characteristic CT features of intussusception include an inhomogeneous “target”- or “sausage”-shaped soft tissue mass with layering effect [11]. Idiopathic intussusception does not generally cause proximal bowel obstruction and possesses distinct anatomic features that can be clearly seen on CT, such as the entering wall, mesenteric fat and vessels, returning wall, and intraluminal space [12, 13]. In this case, because bowel obstruction, tumor, or bowel wall edema was not observed on CT, it was possible to diagnose idiopathic intussusception. More than half of large bowel intussusceptions are associated with malignant lesions [7]. Therefore, confirming the specific features mentioned above on CT is important when deciding the surgical strategy for treatment of adult colo-colonic intussusception.

Laparoscopic surgical reduction for adult idiopathic intussusception was useful and effective. The earliest description of laparoscopic-assisted resection of intussusception due to Meckel’s diverticulum was reported in 1993 [14]. Since then, the number of reports of laparoscopic approach for intussusception had gradually increased. According to previous reports, laparoscopic approach for adult intussusception was more useful than open in the following points: the diagnostic point of view and the short-term clinical outcomes, e.g., earlier oral intake and a lower comprehensive complication index [15], while there might be more serious complications, such as bowel perforation and major vessel injury, in laparoscopic approach [16]. Although patients with intussusception normally present with chronic symptoms and do not usually present with acute intestinal obstruction with significant abdominal distension, early conversion to the open procedure should be considered when necessary.

Surgical reduction of intussusception before resection is not recommended in adults due to the risk of bowel perforation. In addition, if the intussusception is associated with a malignant tumor, reduction may cause tumor cell dissemination [2]. In contrast, the usefulness of laparoscopic surgical reduction in children with intussusception is widely recognized, as most pediatric cases are idiopathic [17–19]. Since not all cases of adult intussusception are associated with tumor, laparoscopic surgical reduction is feasible if idiopathic intussusception is confirmed on preoperative imaging, as illustrated by this case.

We identified relevant studies by searching the PubMed database using the terms “adult idiopathic intussusception” and “colonic”. Only three cases, including this case, were found as true idiopathic intussusception cases with no apparent cause [20, 21]. In two out of three cases, surgery was selected because of strong abdominal symptoms. Cases with few symptoms were treated using non-invasive treatment like endoscopic reduction [21]. Endoscopic approach was very useful because diagnosis and treatment could be performed at the same time. Thus, it is considered to be important to decide the treatment strategy according to the degree of symptoms; in cases with few symptoms, non-invasive reduction, such as endoscopy and barium examination, was a first choice and incases with strong symptoms, surgery was performed with careful consideration for bowel ischemia. Moreover, it is considered necessary to evaluate the mucosal surface for distinguishing the condition from special intussusception, such as parasites [22, 23]. We believe that minimal bowel resection or early endoscopic observation after surgery was desirable.

The reduction success rate by laparoscopy was ≥ 70%, and the success rate was particularly high in ileo-colonic intussusception [17]. The reduction enables avoiding the excessive length of the bowel resection. Surgical reduction methods for adult intussusception are not definitive. These methods involving a combination of delicate direct pressure on the anal side of transverse colon and gentle pulling on the oral side, which is often used for children, may be applicable to adults as in the present case. But, surgical reduction should not be attempted if there are signs of inflammation or ischemia of the bowel wall.

Laparoscopic approach for adult intussusception still has many challenges. When they are limited to adult intussusception without a tumor, laparoscopic approach and surgical reduction can be performed safely. Going forward, as the laparoscopic approach is used more frequently for all types of intussusceptions, its use for acute abdominal conditions will continue to expand.

## Data Availability

Data sharing is not applicable to this article as no datasets were generated or analyzed during the study.

## Abbreviation

CT: Computed tomography

## References

[1] Azar T, Berger DL (1997). Adult intussusception. Ann Surg.

[2] Honjo H, Mike M, Kusanagi H, Kano N (2015). Adult intussusception: a retrospective review. World J Surg.

[3] Lianos G, Xeropotamos N, Bali C, Baltoggiannis G, Ignatiadou E (2013). Adult bowel intussusception: presentation, location, etiology, diagnosis and treatment. G Chir.

[4] Edwards EA, Pigg N, Courtier J, Zapala MA, MacKenzie JD, Phelps AS (2017). Intussusception: past, present and future. Pediatr Radiol.

[5] Martín-Lorenzo JG, Torralba-Martinez A, Lirón-Ruiz R, Flores-Pastor B, Miguel-Perelló J, Aguilar-Jimenez J (2004). Intestinal invagination in adults: preoperative diagnosis and management. Int J Colorectal Dis.

[6] Palanivelu C, Rangarajan M, Senthilkumar R, Madankumar MV (2007). Minimal access surgery for adult intussusception with subacute intestinal obstruction: a single center’s decade-long experience. Surg Laparosc Endosc Percutan Tech.

[7] Valentini V, Buquicchio GL, Galluzzo M, Ianniello S, Di Grezia G, Ambrosio R (2016). Intussusception in adults: the role of MDCT in the identification of the site and cause of obstruction. Gastroenterol Res Pract.

[8] Huang BY, Warshauer DM (2003). Adult intussusception: diagnosis and clinical relevance. Radiol Clin North Am.

[9] Takeuchi K, Tsuzuki Y, Ando T, Sekihara M, Hara T, Kori T (2003). The diagnosis and treatment of adult intussusception. J Clin Gastroenterol.

[10] Gayer G, Zissin R, Apter S, Papa M, Hertz M (2002). Pictorial review: adult intussusception—a CT diagnosis. Br J Radiol.

[11] Gayer G, Apter S, Hofmann C, Nass S, Amitai M, Zissin R (1998). Intussusception in adults: CT diagnosis. Clin Radiol.

[12] Kim YH, Blake MA, Harisinghani MG, Archer-Arroyo K (2006). Adult intestinal intussusception: CT appearances and identification of a causative lead point. Radiographics.

[13] Saw EC, Ramachandra S (1993). Laparoscopically assisted resection of intussuscepted Meckel’s diverticulum. Surg Laparosc Endosc.

[14] Li Siow S, Qiang Goo Z, Mahendran HA, Wong CM (2019). Laparoscopic versus open management of adult intussusception. Surg Endosc.

[15] Kang S, Lee SI, Min BW, Lee TH, Baek S-J, Kwak J-M (2020). A multicenter comparative study between laparoscopic and open surgery for intussusception in adults. Colorectal Dis.

[16] Apelt N, Featherstone N, Giuliani S (2013). Laparoscopic treatment of intussusception in children: a systematic review. J Pediatr Surg.

[17] Sklar CM, Chan E, Nasr A (2014). Laparoscopic versus open reduction of intussusception in children: a retrospective review and meta-analysis. J Laparoendosc Adv Surg Tech A.

[18] Guo W, Hu Z, Tan Y-I, Sheng M, Wang J (2017). Risk factors for recurrent intussusception in children: a retrospective cohort study. BMJ Open.

[19] Sato M, Hamada Y, Fukuda H (1997). Laparoscopic reduction of intussusception. Pediatr Endosurg Innov Tech.

[20] Michele A, D’Abbicco D, Stefania P, Angelica C, Antonio M (2013). Idiopathic adult colo-colonic intussusception: case report and review of the literature. Int J Surg Case Rep.

[21] Mitsunobu I, Ho MK, Shigeyoshi H, Jun K, Hisanori H, Koichi D (2019). Laparoscopic surgery for idiopathic adult intussusception successfully reduced by colonoscopy. J Anus Rectum Colon.

[22] Tanwar R, Jain SK, Bains L (2014). Amoebic colitis presenting as ileocaecal intussusception—a rare case. Malays J Med Sci.

[23] Pham T, La Paglia D, Pitcher M (2017). Salmonella enteritis: a rare cause of adult intussusception. Ann Coloproctol.

